# A clinical decision support system learned from data to personalize treatment recommendations towards preventing breast cancer metastasis

**DOI:** 10.1371/journal.pone.0213292

**Published:** 2019-03-08

**Authors:** Xia Jiang, Alan Wells, Adam Brufsky, Richard Neapolitan

**Affiliations:** 1 Department of Biomedical Informatics, University of Pittsburgh, Pittsburgh, Pennsylvania, United States of America; 2 Department of Pathology, University of Pittsburgh and Pittsburgh VA Health System, Pittsburgh, Pennsylvania, United States of America; 3 UPMC Hillman Cancer Center, University of Pittsburgh School of Medicine, Pittsburgh, Pennsylvania, United States of America; 4 Division of Hematology/Oncology, University of Pittsburgh School of Medicine, Pittsburgh, Pennsylvania, United States of America; 5 Department of Preventive Medicine, Northwestern University Feinberg School of Medicine, Chicago, Illinois, United States of America; Gangnam Severance Hospital, Yonsei University College of Medicine, REPUBLIC OF KOREA

## Abstract

**Objective:**

A *Clinical Decision Support System* (*CDSS*) that can amass *Electronic Health Record* (*EHR*) and other patient data holds promise to provide accurate classification and guide treatment choices. Our objective is to develop the *D**ecision Support System for Making* *P**ersonalized* *A**ssessments and Recommendations Concerning Breast* *C**ancer Patients* (*DPAC*), which is a CDSS learned from data that recommends the optimal treatment decisions based on a patient’s features.

**Method:**

We developed a Bayesian network architecture called *Causal Modeling with Internal Layers* (*CAMIL*), and an algorithm called *Treatment Feature Interactions* (*TFI*), which learns from data the interactions needed in a CAMIL model. Using the TFI algorithm, we learned interactions for six treatments from the LSDS-5YDM dataset. We created a CAMIL model using these interactions, resulting in a DPAC which recommends treatments towards preventing 5-year breast cancer metastasis.

**Results:**

In a 5-fold cross-validation analysis, we compared the probability of being metastasis free in 5 years for patients who made decisions recommended by DPAC to those who did not. These probabilities are (the probability for those making the decisions appears first): chemotherapy (.938, .872); breast/chest wall radiation (.939, .902); nodal field radiation (.940, .784); antihormone (.941, .906); HER2 inhibitors (.934, .880); neadjuvant therapy (.931, .837). In an application of DPAC to the independent METABRIC dataset, the probabilities for chemotherapy were (.845, .788).

**Discussion:**

Patients who took the advice of DPAC had, as a group, notably better outcomes than those who did not. We conclude that DPAC is effective at amassing and analyzing data towards treatment recommendations. Some of the findings in DPAC are controversial. For example, DPAC says that chemotherapy increases the chances of metastasis for many node negative patients. This controversy shows the importance of developing a conclusive version of DPAC to ensure we provide patients with the best patient-specific treatment recommendations.

## Introduction

*Precision medicine* promises to help us improve patient outcomes by tailoring healthcare to the individual patient. Researchers have made tremendous efforts in using results learned from “-omics” data to guide the development and selection of effective drugs for patients. However, the *electronic health record* (*EHR*) database, a widely available resource, has been underutilized for the purpose of tailoring therapies. An EHR database contains abundant data about patients’ clinical features, disease status, interventions, and clinical outcomes, affording us the opportunity to provide highly-personalized medicine beyond only looking at the genomic level [1]. It is believed that, “coupled with new analytics tools, they open the door to mining information for the most effective outcomes across large populations” [2]. Such data are invaluable to tailoring treatments to individuals with diseases such as cancer and Alzheimer’s disease.

Breast cancer is the most common cancer in women. Clinicians face hard decisions in many aspects of breast cancer. For example, image-guided core needle biopsy of the breast is a common procedure that can be non-definitive in 5%-15% of women. This makes it difficult to further classify breast cancer into subtypes. Variation in breast cancer subtypes has been known to be associated with a patient’s drug response, progression of the tumor, and survival of the patient [3,4]. There is also great uncertainty in the treatment and prognosis for breast cancer. For example, HER2-amplified breast cancer is a subtype with poor prognosis if untreated, but the targeted therapeutic trastuzumab (Herceptin) has vastly improved the survival of such patients. Although Herceptin is used in the therapy of all patients with HER2-amplified tumors, only some respond. Furthermore, it is expensive and can cause cardiac toxicity [2]. So, it is important to give it only to patients who benefit from it. Other studies show that thousands of genes are associated with subtype and prognosis of breast cancer, and particular allele combinations may usefully guide the treatment selection [5]. A *Clinical Decision Support System* (*CDSS*) that can amass all this genomic information and combine it with clinical information holds promise to provide accurate classification and guide treatment choices.

We develop a Bayesian network architecture called *Causal Modeling with Internal Layers* (*CAMIL*), and an algorithm called *Treatment Feature Interactions* (*TFI*), which learns from data the interactions needed in a CAMIL model. We transform the CAMIL model to an influence diagram, resulting in a probability-based CDSS called *D**ecision Support System for Making* *P**ersonalized* *A**ssessments and Recommendations Concerning Breast* *C**ancer Patients* (*DPAC*), which recommends treatment decisions based on a patient’s features. The DPAC, which we develop in this paper, recommends six treatment decisions to prevent 5-year breast cancer metastasis, and is learned from the *Lynn Sage Data Set with 5-Year Distant Metastasis* (*LSDS-5YDM*), which we curated. We evaluate DPAC using a 5-fold cross-validation analysis, and by applying the DPAC learned from the entire LSDS-5YDM to the independent METABRIC breast cancer dataset [6]. In these evaluations, we compare the outcomes of patients who made the treatment decision recommended by DPAC to the outcomes of ones who did not make that decision. In both cases, patients who took the treatment advice of DPAC had, as a group, notably better outcomes than those who did not.

## Method

Since DPAC is based on Bayesian networks and influence diagrams, we first review these.

### Bayesian networks

**Fig 1:**
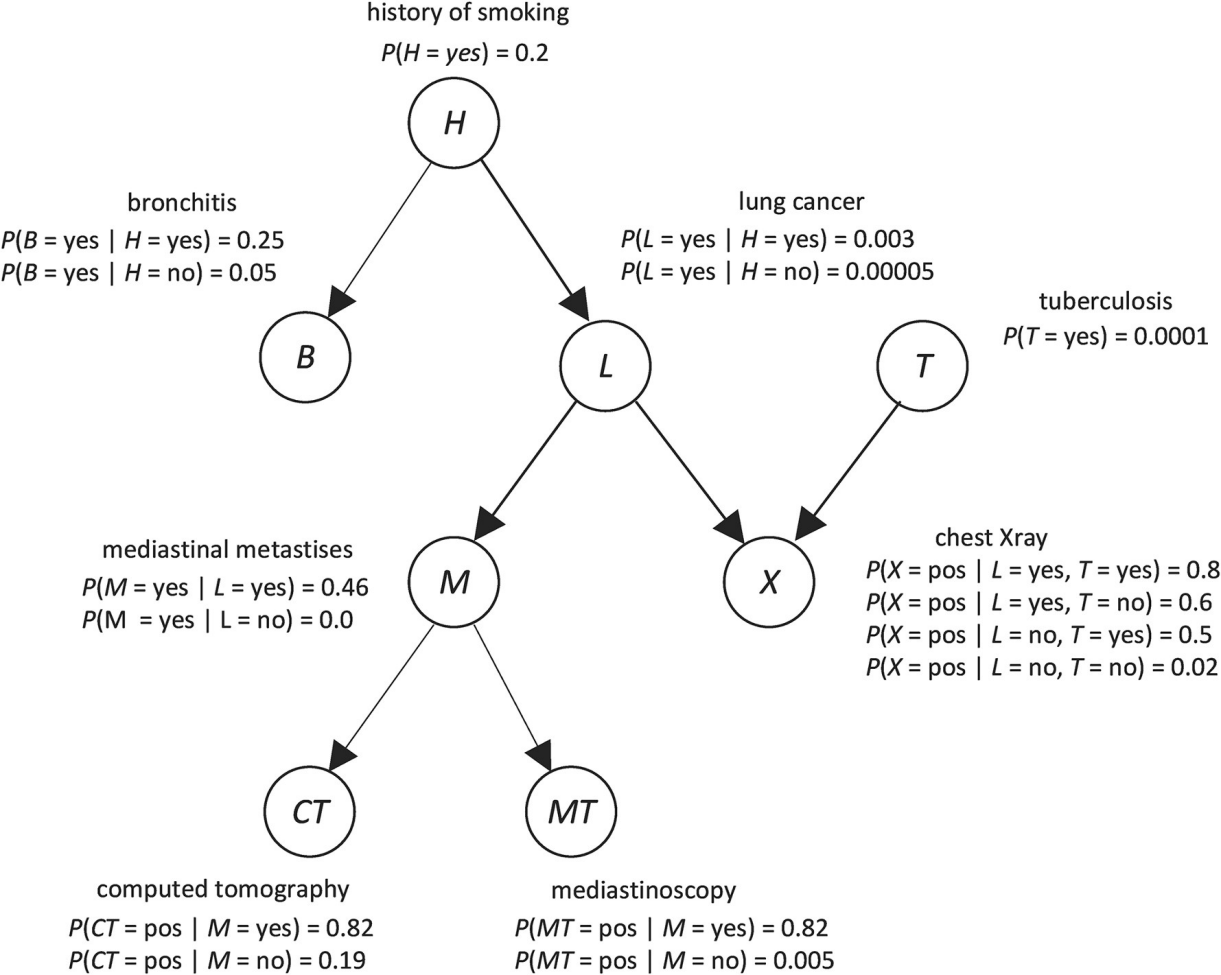
A Bayesian network.

*Bayesian networks* (*BN*) are a leading architecture for handling uncertainty in artificial intelligence and machine learning [7–10]. A BN consists of a *directed acyclic graph* (*DAG*), whose edges represent direct probabilistic dependencies; the prior probability distribution of every variable that is a root in the DAG; and the conditional probability distribution of every non-root variable given each set of values of its parents. Fig 1 shows a BN modeling relationships among variables related to respiratory diseases. The node *H*, which represents “history of smoking”, is a root. Since it is a root, the BN contains its prior probability distribution, which is the probability of an individual having a history of smoking given that the individual is from the population we are investigating. Assuming 20% of individuals in the population have smoked, we assign *P*(*H* = yes) = 0.2, and *P*(*H* = no) = 0.8. The node *L*, which represents “lung cancer”, is not a root; so the BN contains its conditional probability distribution given each value of its parent *H*. Assuming 0.3% of smokers in the population get lung cancer, we assign *P*(*L* = yes |*H* = yes) = 0.003. Assuming 0.005% of nonsmokers in the population get lung cancer, we assign *P*(*L* = yes |*H* = no) = 0.00005.

Using a BN, we can determine probabilities of interest with a BN inference algorithm [7]. For example, with the BN in Fig 1, if a patient has a smoking history (*H* = yes), positive chest X-ray (*X* = pos), and positive computer tomography (*CT* = pos), we can determine the probability of the patient having lung cancer (*L* = yes). That is, we can compute *P*(*L* = yes| *H* = yes, *X* = pos, *CT* = pos), which turns out to be 0.185.

Learning a BN from data concerns learning both the parameters and the structure (called a DAG model). In the score-based structure-learning approach, a score is assigned to a DAG model *G* based on how well *G* fits the *Data*. The Bayesian score [11] is the probability of the *Data* given DAG model *G*. For discrete variables, this score often uses a Dirichlet distribution to represent prior belief for each conditional distribution in *G*. The score is as follows:

scoreBayes(G:Data)=P(Data|G)=∏i=1n∏j=1qiΓ(∑k=1riaijk)Γ(∑k=1riaijk+∑k=1risijk)∏k=1riΓ(aijk+sijk)Γ(aijk),

where *n* is the number of variables, *r*_*i*_ is the number of states of *X*_*i*_, *q*_*i*_ is the number of different values that the parents of *X*_*i*_ can jointly assume, *a*_*ijk*_ is a hyperparameter, and *s*_*ijk*_ is the number of times *X*_*i*_ took its *k* th value when the parents of *X*_*i*_ took their *j* th value. When *a*_*ijk*_ = *α* / *r*_*i*_*q*_*i*_ for a parameter *α*, the score is called the *Bayesian Dirichlet equivalent uniform* (*BDeu*) score [12]. The BN model selection problem is NP-hard [13]. So, heuristic search algorithms are often employed [7].

### Influence diagrams

**Fig 2:**
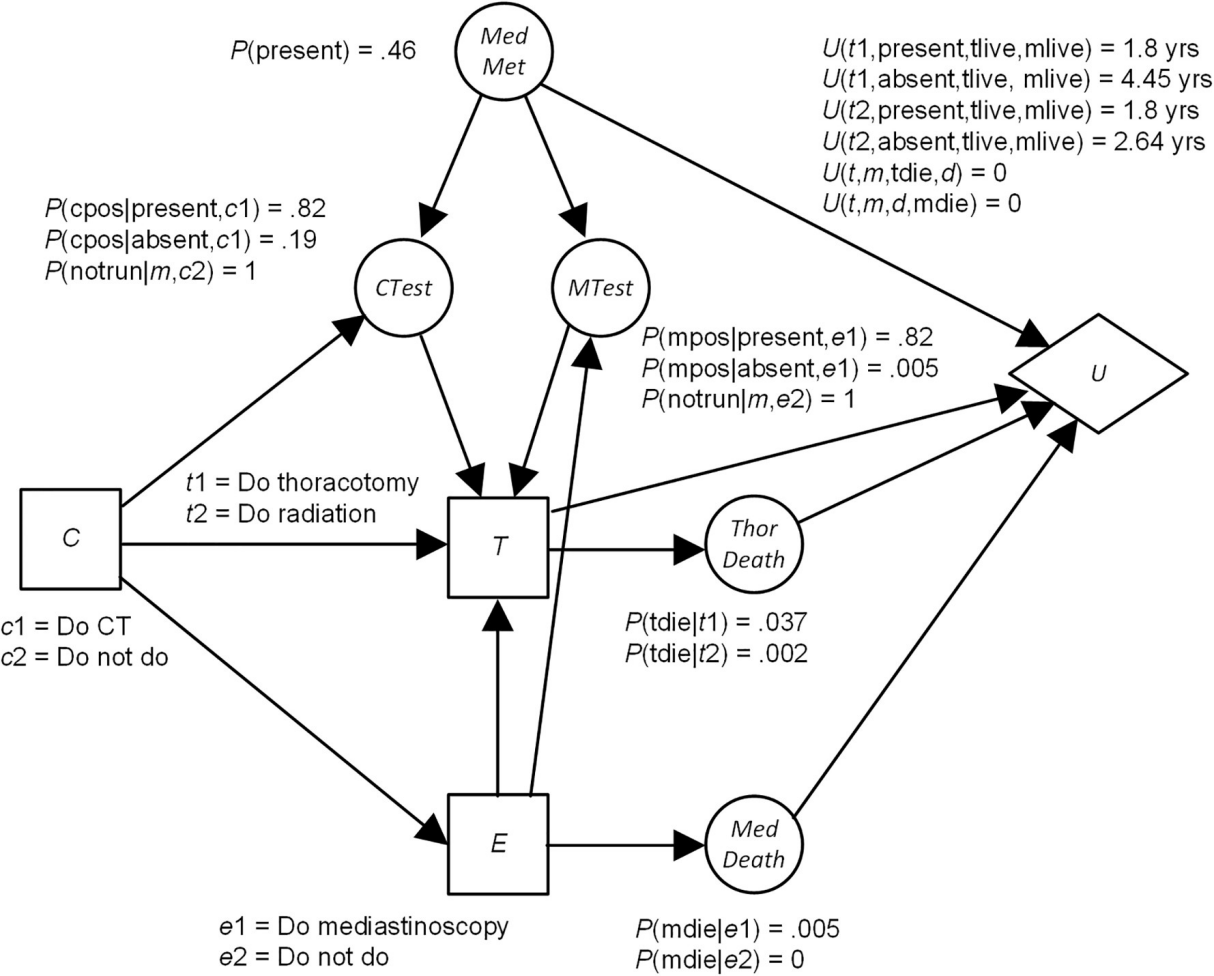
An influence diagram.

An *influence diagram* (*ID*) is a BN augmented with decision nodes and utility nodes. An ID not only provides us with a joint probability distribution, but also recommends decisions based on the patient’s preferences. Fig 2 shows an ID modeling the decision of whether or not to be treated with a thoracotomy for a non-small-cell carcinoma of the lung, based on an ID in [14]. The nodes with prior probabilities in Fig 2 are chance nodes, as in BNs. The rectangular nodes are decision nodes. An edge into a decision node is an information edge and represents what is known when the decision is made. The hexagon node is a utility node, and represents the utility of the outcomes to the patient. Edges into this node represent features that directly affect this utility.

Algorithms for solving IDs determine the decision alternative for the first decision that maximizes expected utility [7]. For the ID in Fig 2, that decision is *C*, which is whether to have a CT scan. In this case the expected utility is the expected life span. It turns out that for *c*1 the expected life span is 3.23 and for *c*2 it is 2.76. So the recommendation is *c*1, which is to have the CT scan.

The utility of the outcome to the patient is often in terms of *quality adjusted life years* (*QALY*) [15] instead of simply years because some treatments and conditions significantly decrease the quality of life. When we use QALY, a year in which the patient is in perfect health has value 1, being dead has value 0, and a non-well year has some value between 0 and 1. For example, a year with a sore throat is estimated to have a value of 0.9 [7]. We can assess QALYs following the guidelines in [7]. When we compute life expectancy using a time-trade-off quality adjustment, we call it quality adjusted life expectancy (QALE). We ordinarily use QALYs instead of simply life years as the utilities in a decision model concerning a treatment or surgery decision, and we make the decision that maximizes QALE.

### Development of DPAC

A DPAC system is developed by learning treatment-feature interactions, incorporating the interactions into a *Causal Modeling with Internal Layers* (*CAMIL*) model, and then converting the CAMIL model to an influence diagram. We discuss each of these in turn.

In order to determine optimal treatment choices, we need to model how treatments interact with patient features to affect outcomes. For example, Herceptin is a treatment for breast cancer which is effective only for HER2+ patients; so Herceptin interacts with HER2 status to affect metastasis. We create a new algorithm that learns such interactions from data.

First, we need a definition of a treatment-feature interaction. The definition is first motivated by an example. Suppose

$$\begin{array}{c} P(\text{metastasis}|\text{HER}2+,\text{Herceptin})=0.01\\ P(\text{metastasis}|\text{HER}2+,\text{No}\;\text{Herceptin})=0.5 \end{array}$$

$$\begin{array}{c} P(\text{metastasis}|\text{HER}2-,\text{Herceptin})=0.3\\ P(\text{metastasis}|\text{HER}2-,\text{No}\;\text{Herceptin})=0.02 \end{array}$$

Then the use of Herceptin is strongly indicated for HER2+ patients and strongly contraindicated for HER2- patients. We would say there is a strong interaction between Herceptin and HER2 status. On the other hand, suppose

$$\begin{array}{c} P(\text{metastasis}|\text{ER}+,\text{Herceptin})=0.05\\ P(\text{metastasis}|\text{ER}+,\text{No}\;\text{Herceptin})=0.05 \end{array}$$

$$\begin{array}{c} P(\text{metastasis}|\text{ER}-,\text{Herceptin})=0.03\\ P(\text{metastasis}|\text{ER}-,\text{No}\;\text{Herceptin})=0.03 \end{array}$$

In this case, we would say there is no interaction between Herceptin and ER status. The Hellinger distance quantifies the degree of dissimilarity between two probability distribution [16]. Its value is 0 if they are the same probability distribution and 1 if they are maximally dissimilar. For a given binary feature and binary treatment, we first compute the Hellinger distance between the probability distribution of the target given the patient has the first value of the feature and had the treatment and the probability distribution of the target given the patient has the first value of the feature and did not have the treatment. We then compute the Hellinger distance between the probability distribution of the target given the patient has the second value of the feature and had the treatment and the probability distribution of the target given the patient has the second value of the feature and did not have the treatment. If the treatment is effective for one value of the feature, and deleterious for the other value of the feature, we take the strength to be the minimum of the two Hellinger distances; otherwise we take it to be 0.

We are assuming that a given treatment is deleterious for some value of a feature with which it interacts because our preliminary investigations showed that this the case. For example, we found that chemotherapy increase the likelihood of 5-year metastasis for node-negative patients with low grade tumors. Our goal here is only to show the effectiveness of our method based on assuming the correctness of our knowledge. So, we are keeping the utility as simple as possible. In application, we would not require that a treatment be deleterious for one value of the feature, but rather we would add a utility node for the negative effect of the treatment on quality of life. If the treatment had a positive effect for a given set of values of the patient’s features, the treatment decision would be based on weighing the benefit of the treatment for avoiding metastasis against the negative effect of the treatment on quality of life. In the next section we will show how quality of life can be considered in the treatment decision.

**Fig 3:**
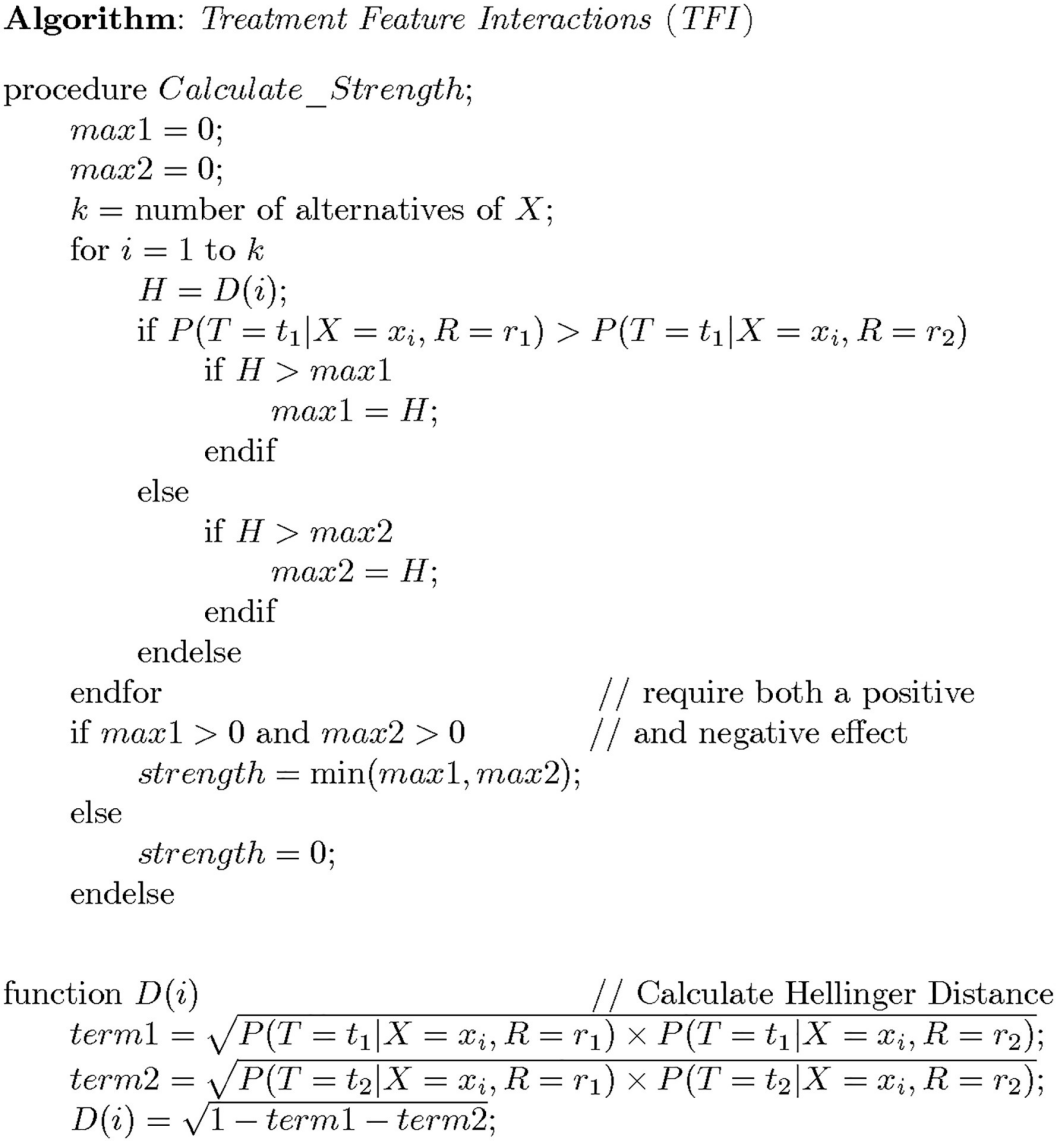
Algorithm TFI, which determines the strength with which binary treatment *R* interacts with variable *X* to affect binary target *T*.

In general, a feature is not binary. So, we take the maximum Hellinger distance over all values of the feature for which the treatment is effective, the maximum Hellinger distance over all values of the feature for which the treatment is ineffective, and take the strength to be the minimum of these two maximums. Fig 3 shows the resultant algorithm, called *Treatment Feature Interactions* (*TFI*), when we are considering only a single feature. However, a given treatment can interact with several variables simultaneously. The algorithm in Fig 3 can be applied where *X* represents a set of variables. In this research we apply it to sets containing 1, 2, 3, and 4 variables.

After applying Algorithm TFI to each treatment, we will have sets containing treatments and features. To develop a prediction algorithm using these sets, we use a model that assumes each set affects the target independently. Our model, called CAMIL, is a generalization of the Noisy-OR model. So, we first review that model.

**Fig 4:**
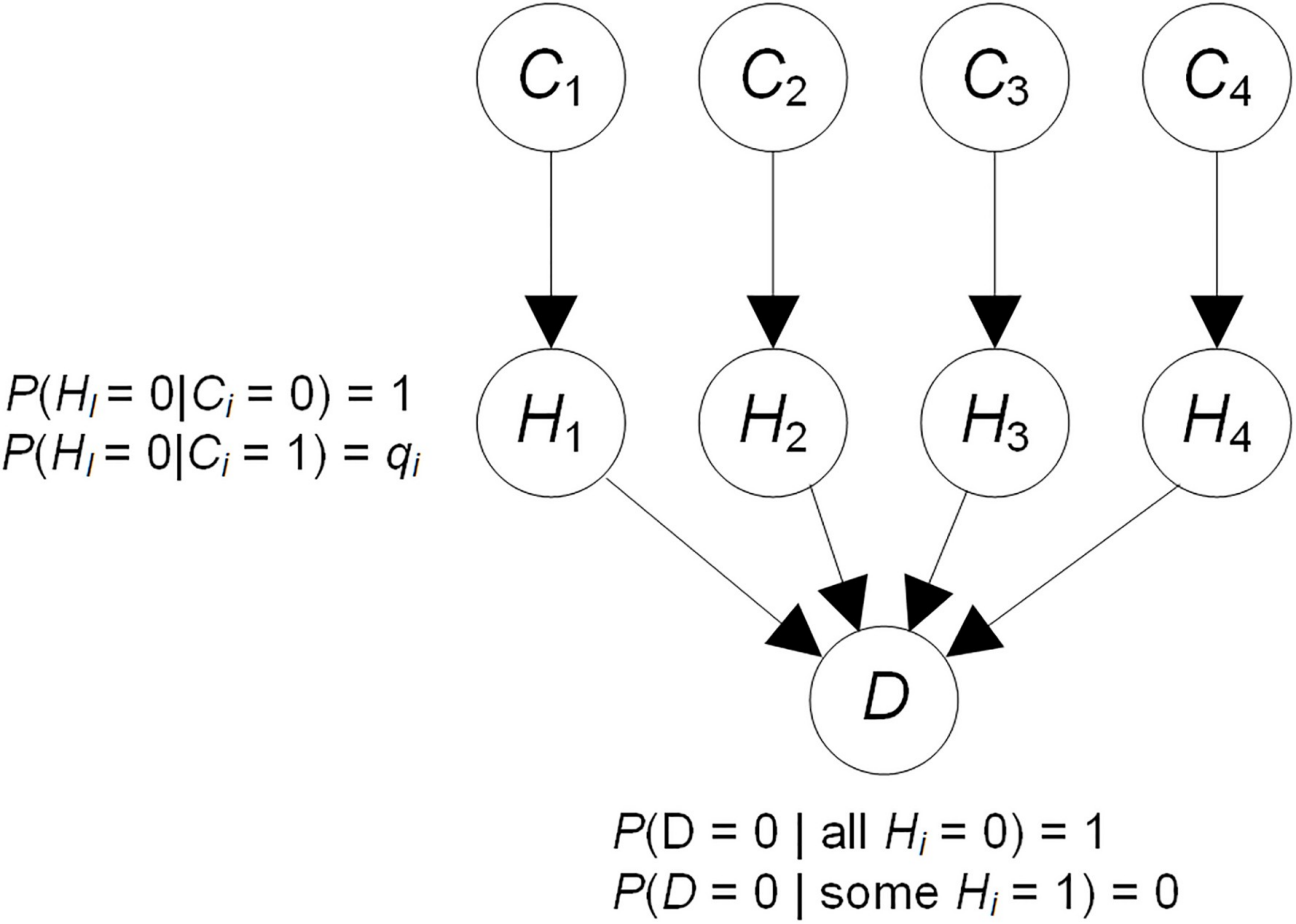
The Noisy-Or model.

The Noisy-Or Bayesian network model [17] concerns binary causes that independently cause a binary target. An example of the Noisy-Or model appears in Fig 4. In this example, there are 4 causes *C*_1_, *C*_2_, *C*_3_, and *C*_4_, of a disease *D*. The variables labeled *H*_*i*_ are hidden. The value 1 represents that the cause is present and the value 0 represents that it is absent. Similarly, the value 1 represents that the disease *D* is present and the value 0 represents that it is absent. The model assumes that the presence of each cause *C*_*i*_ will result in *D* being present, regardless of the presence of the other causes, unless *C*_*i*_ is inhibited. Cause *C*_*i*_ has probability *q*_*i*_ of being inhibited when it has value 1. The value of 1 − *q*_*i*_ is called the *causal strength* of *C*_*i*_ for *D*. It is possible to show that

$$P(D=0|C_{1},\;C_{2},\;C_{3},\;C_{4})=\underset{i\;:\;C_{i}=1}{\prod }q_{i}.$$

So once we know the values of *q*_*i*_, it straightforward to compute the probability of *D* given any combination of the causes. To estimate the value of *q*_*i*_ we can set

$$q_{i}=\frac{a_{0}}{a_{0}+a_{1}},$$ (1)

where *a*_0_ is the number of records in which *C*_*i*_ = 1, all other *C*_*j*_ = 0, and *D* = 0; and *a*_1_ is the number of records in which *C*_*i*_ = 1, all other *C*_*j*_ = 0, and *D* = 1.

If we had sufficient data, we could use Eq 1 to learn the parameters for the Noisy-Or model. However, if there are many predictors and the dataset is not extremely large, the values of *a*_0_ and *a*_1_ will be very small and often even zero for many predictors. So, we use an *Expectation Maximization* (*EM*) [7] algorithm to learn parameters. By using an EM algorithm we are able to learn something about *q*_*i*_ from records that do not have *C*_*i*_ equal to 1 and all other *C*_*j*_ equal to 0.

The *leaky Noisy-Or model* assumes that all causes that have not been articulated can be grouped into one hidden cause *Leak*. The hidden cause *Leak* is at the same level as the other hidden variables (the *H*_*i*_) but it has no parents. We have that

$$P(Leak=0)=q_{Leak}.$$

An EM algorithm can learn *q*_*Leak*_ along with the other parameters.

**Fig 5:**
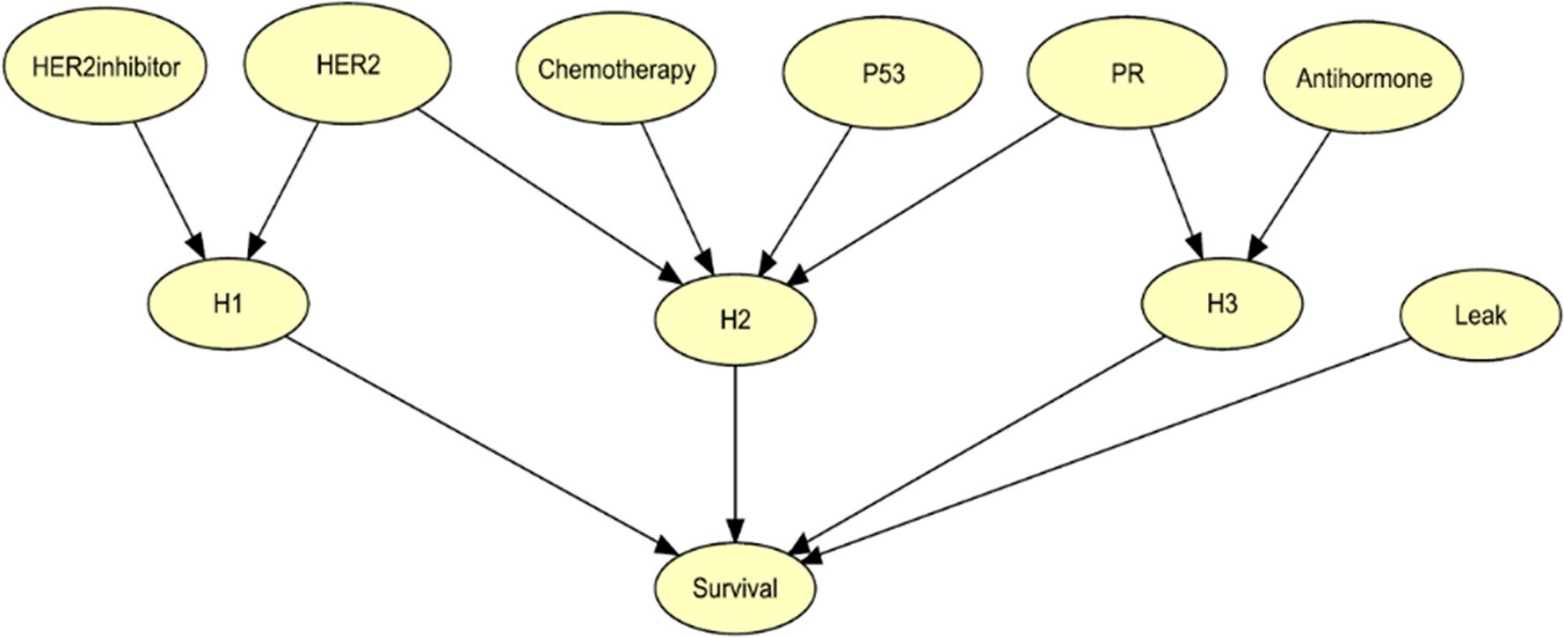
An example of a CAMIL model. This model is only for illustration. It was not learned from data.

CAMIL is an extension of the Leaky Noisy-Or Model with the following additional features: 1) The causes may be non-binary; and 2) causes may interact. CAMIL assumes the interactions independently affect the target according to the assumptions in the Leaky Noisy-Or Model. Fig 5 shows an example in which three clinical features (*HER2*, *P53*, and *PR*) and three treatments (*HER2inhibitor*, *Chemotherapy*, and *Antihormone*) are the causes and the target is *Survival*. This network was not learned from data, but rather is only for illustration. The variables labeled *H*_*i*_ are hidden binary variables. There is a single hidden variable for each interaction and each non-interacting cause. According to the model in Fig 5, *HER2inhibitor* and *HER2* interact. So they are parents of a hidden variable. Similarly, *Chemotherapy* interacts with all three clinical variables. So they are all parents of a hidden variable. The *Leak* variable is also hidden, and represents causes not identified in the model.

If the parent variables of a hidden variable *H*_*i*_ have *k* values, there are *k* conditional distributions for *H*_*i*_. For example, if *H*_*i*_ has one cause *C*_*i*_, the distributions are as follows:

$$\begin{array}{ll} q_{i1}= & P(H_{i}=0|C_{i}=1)\\ q_{i2}= & P(H_{i}=0|C_{i}=2)\\ & \vdots \\ q_{ik}= & P(H_{i}=0|C_{i}=k) \end{array}$$

An EM algorithm can learn the parameter values in a CAMIL model.

It is possible to extend the CAMIL model to a non-binary target as long as the target’s values are on an ordinal scale. That is, the target has increasingly strong values such as low, medium, and high. For example, if these are the three values of the target, we give each hidden parent variable of the target values 1, 2, and 3. If any parent has value 3, the target has value high; if any parent has value 2 and no parent has value 3, the target has value medium; if no parent has value 2 or 3, the target has value low.

Once we learn a CAMIL model based on interactions between treatments and features, it is straightforward to convert it to an influence diagram. First, we make each treatment node a decision node. Then we create a value node as a child of each outcome node. For example, if the outcome is simply 5-year survival, we can create a value node that has value 1 if the patient survives and value 0 otherwise. We call the resultant system DPAC.

### Datasets

The *Lynn Sage Data Base* (*LSDB*) contains information about patients who came to the Lynn Sage Comprehensive Breast Center at Northwestern Memorial Hospital for care. The *Northwestern Medicine Enterprise Data Warehouse* (*NMEDW*) is a joint initiative across the Northwestern University Feinberg School of Medicine and Northwestern Memorial HealthCare, which maintains comprehensive data obtain from EHRs. Using the LSDB and the NMEDW, we curated a dataset named as the *Lynn Sage Data Set* with 5-year distant metastasis (*LSDS-5YDM*), which includes records on 6726 breast cancer patients including clinical features and distant metastasis. The records span 03/02/1990 to 07/28/2015. Table 1 shows the clinical features and treatments in the LSDS-5YDM that are included in this study, and their values.

**Table 1:** The variables in the LSDS-5YDM that are included in this study.

Variable	Description	Values
Age	age at diagnosis of the disease	0–49, 50–64, > 64
menopause	inferred menopausal status	pre, post
size	size of tumor in mm	0–38, 38–50.5, > 50.5
node_positive	number of positive lymph nodes	0, 1–3, > 3
node_removed	number of lymph nodes removed	0–2, 3–5 > 5
node_status	patient had any positive lymph nodes	neg,pos
grade	grade of disease	1, 2, 3
invasive	whether tumor is invasive	yes,no
stage	composite of size and # positive nodes	0,1,2,3
histology	tumor histology	lobular, duct
ER	estrogen receptor expression	neg, pos
PR	progesterone receptor expression	neg, pos
HER2	HER2 expression	neg, pos
TNEG	patient ER, PR, and HER2 negative	yes, no
P53	whether P53 is mutated	neg, pos
surgical_margins	Whether there is a residual tumor after surgery	res. tumor, no res. tumor, no primary site surgery
surgery	type of surgery	conservation, mastectomy
chemo	whether patient had chemotherapy	yes, no
breast_chest_radi	whether patient had breast or chest radiation	yes, no
nodal_radi	whether patient had lymph node radiation	yes, no
antihormone	whether patient had hormone therapy	yes, no
HER2_Inhib	whether patient had a HER2 inhibitor	yes, no
neo	Whether patient had neoadjuvant therapy	yes, no

We discuss in some detail the variables whose meanings are not straightforward. Histology provides the nature of a tumor. That is, whether it is confined to the breast ducts, which are tubes that carry the milk to the nipple, or whether it has infiltrated the milk producing glands (lobules). If the breast cancer has a significant number of receptors for estrogen or for progesterone, it is considered estrogen receptor (ER) positive or progesterone receptor (PR) positive. HER2-positive breast cancer is cancer that tests positive for a protein called human epidermal growth factor receptor 2 (HER2), which promotes the growth of cancer cells. P53 is a gene that codes for a protein that regulates the cell cycle and functions as a tumor suppression. If the gene is mutated, metastasis prognosis is worse.

We used 5-year metastasis as our target in this study. We assigned the value *yes* to *metastasis* if the patient had evident metastases within 5 years of the initial diagnosis, the value *no* to *metastasis* if it was known that the patient did not present with metastases within 5 years, and the value NULL to *metastasis* if the patient discontinued follow-up within the first five years and without evidence of metastases prior to loss to follow-up. The value NULL was also assigned to all missing data fields in all variables. Missing data were then filled in using the nearest neighbor (NN) imputation algorithm.

The *Molecular Taxonomy of Breast Cancer International Consortium* (*METABRIC*) data set [6] has data on 1989 breast cancer tumors. Features include 21 clinical features, expression levels for 16,384 genes, and breast cancer survival. The only treatment variable in the METABRIC dataset that is comparable to the ones we investigated is chemotherapy.

### Experiments

Using the LSDS-5YDM and Algorithm TFI, we calculated the interaction strength, relative to the target being 5-year metastasis, of each treatment (chemo, breast_chest_radi, nodal_radi, antihormone, HER2_Inhib, neo) with all 1, 2, and 3, and 4 set combinations of other variables. We chose the highest scoring set *S*, and we then verified there were no common causes of the treatment and the target that were not independent of the target given the variables in *S*. We did this by forcing edges from the treatment and the variables in *S* to the target, and then applying the *Greedy Equivalent Search* (*GES*) [7] to learn a BN containing all the variables. If the treatment and target did have a common cause that was not independent of the target given variables in *S*, we rejected *S* and went down to the second highest scoring set. We then created a CAMIL model using the learned interactions where 5-year metastasis is the target, and learned parameter values for that model using the EM algorithm. Note that this algorithm learns the parameters for all the hidden nodes simultaneously, which entails that it takes into account the relative effect of the interactions on the target, and therefore the synergistic effects of the treatments. The CAMIL model was then converted to an ID by converting the nodes representing decisions to decision nodes, and adding a utility node, whose value was 0 if the patient metastasized in 5 years and 1 if she did not. The resultant system is called DPAC.

We did the procedure just described in a 5-fold cross-validation analysis using the LSDS-5YDM. For each fold, a DPAC system was learned from the records in the other 4 folds. This DPAC was then applied to each record in the fold to recommend the five decisions. The following values were recorded for each decision:

n1: The number of subjects who made the recommended decision
k1: The number of subjects who made the recommended decision and did not metastasize
n2: The number of subjects who did not make the recommended decision
k2: The number of subjects who did not make the recommended decision and did not metastasize

The values of n1, k1, n2, and k2 were summed over all 5 folds, yielding summed values N1, K1, N2, K2. The ratio K1/N1 then estimates the average utility if the subject made the recommended decision, which, due to the simplicity of the ID, is also the estimate of the probability of not metastasizing given one makes the recommended decision. The ratio K2/N2 provides the same estimates for subjects who did not make the recommended decision.

We applied a DPAC system, which was learned from the entire LSDS-5YDM data, to the independent METABRIC dataset. The outcome for this analysis was whether the patient died from breast cancer in 5 years because that is what was recorded in this dataset, and the only treatment analyzed was chemotherapy because that is all that was available.

### Ethics statement

This paper is a result of the PROTOCOL TITLE: A New Generation Clinical Decision Support System, which was approved by Northwestern University IRB #: STU00200923-MOD0006.

The need for patient consent was waived by the ethics committee because the data consists only of de-identified data mined from EHR databases.

## Results

Table 2 shows the results of the 5-fold cross-validation analyses. As a group, for every decision those who made the decision recommended by the model had notably better outcomes than those who did not. For every decision, the bottom of the 95% confidence interval for the probability of not metastasizing given one made the recommended decision is above the top of the 95% confidence interval for the probability of not metastasizing given one did not make the recommended decision.

**Table 2:** The result of the 5-fold-cross validation analysis. Row 6 shows the estimate of the probability of not metastasizing given the subjects made the decision recommended by the model, and Row 7 shows 95% confidence interval for that estimate. Rows 8 and 9 show the same values of individuals who did not make the decision recommended by the model.

	chemo	breast_chest_radi	nodal_radi	antihormone	HER2_Inhib	neo
N1	4955	3378	4955	2819	5072	5970
K1	4648	3172	4648	2653	4738	5560
N2	1771	3348	1771	3907	1654	756
K2	1545	3021	1545	3540	1455	633
P(not met | dec)	.938	.939	.940	.941	.934	.931
95% conf. interval	(.931,945)	(.930,.947)	(.934,.946)	(.932,.950)	(.927,.941)	(.925,.938)
P(not met | not dec)	.872	.902	.784	.906	.880	.837
95% conf. interval	(.856,.888)	(.892,.912)	(.754,.811)	(.896,.915)	(.863,.895)	(809,.863)

Table 3 shows the results for chemotherapy when we applied the DPAC system, learned from the entire LSDS-5YDM, to the independent METABRIC dataset. These results are similar to the ones obtained in the 5-fold cross-validation; namely, as a group those who made the decision recommended by the model had better outcomes than those who did not, and notably again with the confidence intervals not overlapping.

**Table 3:** The result of applying the DPAC system, learned from the entire LSDS-5YDM, to the METABRIC dataset. Row 6 shows the estimate of the probability of not metastasizing given the subjects made the decision recommended by the model, and Row 7 shows 95% confidence interval for that estimate. Rows 8 and 9 show the same values of individuals who did not make the decision recommended by the model.

	chemo
N1	1291
K1	1091
N2	698
K2	550
P(not met | dec)	.845
95% conf. interval	(.824,864)
P(not met | not dec)	.788
95% conf. interval	(.756,.818)

**Fig 6:**
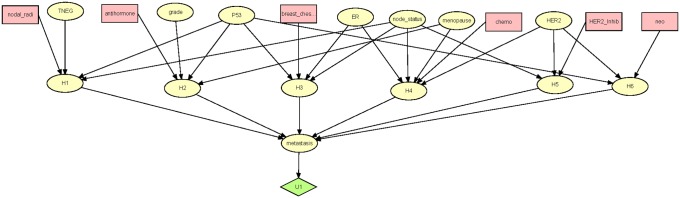
The DPAC system learned from the entire LSDS-5YDM. The Leak node is not shown.

Fig 6 shows the DPAC system learned from the entire dataset. Next we discuss that ID, and, while so doing, provide insight into some of the results in Table 2. Before proceeding, we note that the results may be considered controversial. One limitation that must be acknowledged is that the results follow from the single dataset at a single site, and that upon further analyses of other datasets from distinct clinical practices, the trends may not be replicated. However, the results are not a reflection on the model, and this communication concerns the approach more than the results. Nevertheless, the controversy does serve to demonstrate the importance of pursuing further large scale analyses.

A second limitation of the study is that 5 year follow-up is considered insufficient in most cases of breast cancer. Breast cancers tend to exhibit more extended periods of dormancy than most other carcinomas. In fact, more than half of the recurrences occur more than 5 years after initial diagnosis and intervention [18]. Further, confounding the analyses is that in the US, the majority of early recurrences occur in the subset of triple negative breast cancer (TNBC: ER-/PR-/HER2-), wherein a five-year disease free survival is considered almost like a cure [19].

According to DPAC, HER2 inhibitors interact with HER2 status and node status. According to the parameters for the model (not shown) HER2 inhibitors are effective only for HER2 positive subjects who also are node positive. They are actually deleterious for HER2 negative subjects. The use of HER2 inhibitors in HER2 negative subjects accounts for much of the poor outcomes for subjects who did not take the model’s advice concerning HER2 inhibitors. As noted in the introduction, Herceptin has been shown to confer no benefit to some HER2 positive patients [2]. According to our model and data these are node negative subjects, which are subjects who already have a relatively low probability of showing metastases or recurrences within the five year time period. That is, the prior probability based on our data of metastasizing in 5 years given no additional information is 0.0792 and the prior probability given HER2 positive and node negative is 0.0513.

Chemotherapy interacts with node status, ER status, HER2 status, and menopause. According to DPAC, it is effective for all node positive subjects. However, it is effective only for node negative subjects who are pre-menopausal and both ER and HER2 positive or both ER and HER2 negative (this last group is mainly the aggressive TNBC subtype). Previous research has shown that HER2 and ER interact to affect metastasis [20], and that the combination of ER negative and HER2 negative has the worst prognosis [21,22]. So, for the most part our results indicate that chemotherapy is beneficial only to the more severe cases. A recent study [23] has indicated that chemotherapy provides no benefit to subjects who are node negative, ER positive, HER2-negative, and had a midrange 21-gene recurrence score by Oncotype Dx, although some benefit of chemotherapy was found in some women 50 years of age or younger. These results are consistent with ours. The use of chemotherapy in these subjects accounts for much of the poor outcomes for subjects who did not take the model’s advice to not have chemotherapy.

Nodal field radiation interacts with node status, TNEG status, and P53 status. It was found to be effective only for subjects who are node positive, TNEG yes (TNBC subtype), and P53 positive, which are the most arduous cases. On the other hand, breast-chest wall radiation, which interacts with lymph node status, P53 status, and ER status, was found to be effective for almost all subjects. It was ineffective in subject who were lymph node positive, P53 positive, and ER positive. Similarly, antihormone therapy, which interacts with menopause status, P53 status, and grade, was effective for almost all subjects. It was ineffective for pre-menopausal subjects who were P53 positive, and had grade 1 tumors. Note that ER status is not in the interaction. A separate analysis using the LSDS-5YDM showed that antihormone therapy is effective in both ER positive and ER negative subjects. Manna and Holz [24] discuss that tamoxifen may be beneficial to ER negative patients, as ER status is based on a quantitative level cut-off and not a genetic deletion or epigenetic silencing of ER. The failure to use breast-chest wall radiation and antihormone therapy accounts for much of the poor outcomes for subjects who did not take the model’s advice concerning the use of these two treatments.

Neoadjuvant therapy interacts with P53 and HER2 status, and was found to be beneficial in terms of reduced metastases only for patients who were P53 negative and HER2 positive.

## Adjustment for quality of life

We developed a DPAC system whose only consideration was to minimize the chances of 5-year metastasis. However, chemotherapy and HER2 inhibitors have significant negative ramifications on overall health and quality of life. If chemotherapy only reduces the chances of 5-year metastasis by 1%, a given patient may prefer not to have it. Previously, we mentioned the use of QALYs. When using QALYs, a year in which the patient is in perfect health has value 1, being dead has value 0, and a year on some treatment that diminishes quality of life has some value between 0 and 1. This approach does not lend itself very well to chemotherapy and HER2 inhibitors because both therapies can have negative side effects that last well beyond the time they are taken. For example, chemotherapy can cause peripheral neuropathy which can last for years, and HER2 inhibitors can cause cardiac toxicity. So, we feel a better approach is to use the *standard gamble* technique. When using this technique, we first carefully inform the patient of all the negative ramifications of chemotherapy. Then we ask her the following question:

Suppose with chemotherapy you have a guarantee of being metastasis free in 5 years, and without it you have a probability *p* of being metastasis free in 5 years. What is the smallest value of *p* for which you would forgo having chemotherapy?

We help the patient, say Mary, determine her personal value of *p* as follows. If *p* = 1, this means Mary would have to be certain she would not metastasize in 5 years to forgo chemotherapy, and therefore if chemotherapy provided any chance of decreasing 5-year metastasis, she would choose chemotherapy. It is likely that Mary would not require certainty to forgo chemotherapy. If she does not require certainty, we ask her about p = 0.5. If *p* = 0.5 then Mary would forgo chemotherapy if her chance of not metastasizing without it was at least 0.5. Mary may not forgo chemotherapy if she only had a 0.5 chance of not metastasizing without it. If she says 0.5 is too small to forgo chemotherapy, we ask her about *p* = 0.75. Using this binary cut approach, we can continue to offer Mary values of *p*, and eventually arrive at her personal value of *p*. If it turns out that *p* = 0.9 for her, then Mary is saying that she would forgo chemotherapy if her chances of not metastasizing without it were at least 0.9.

**Fig 7:**
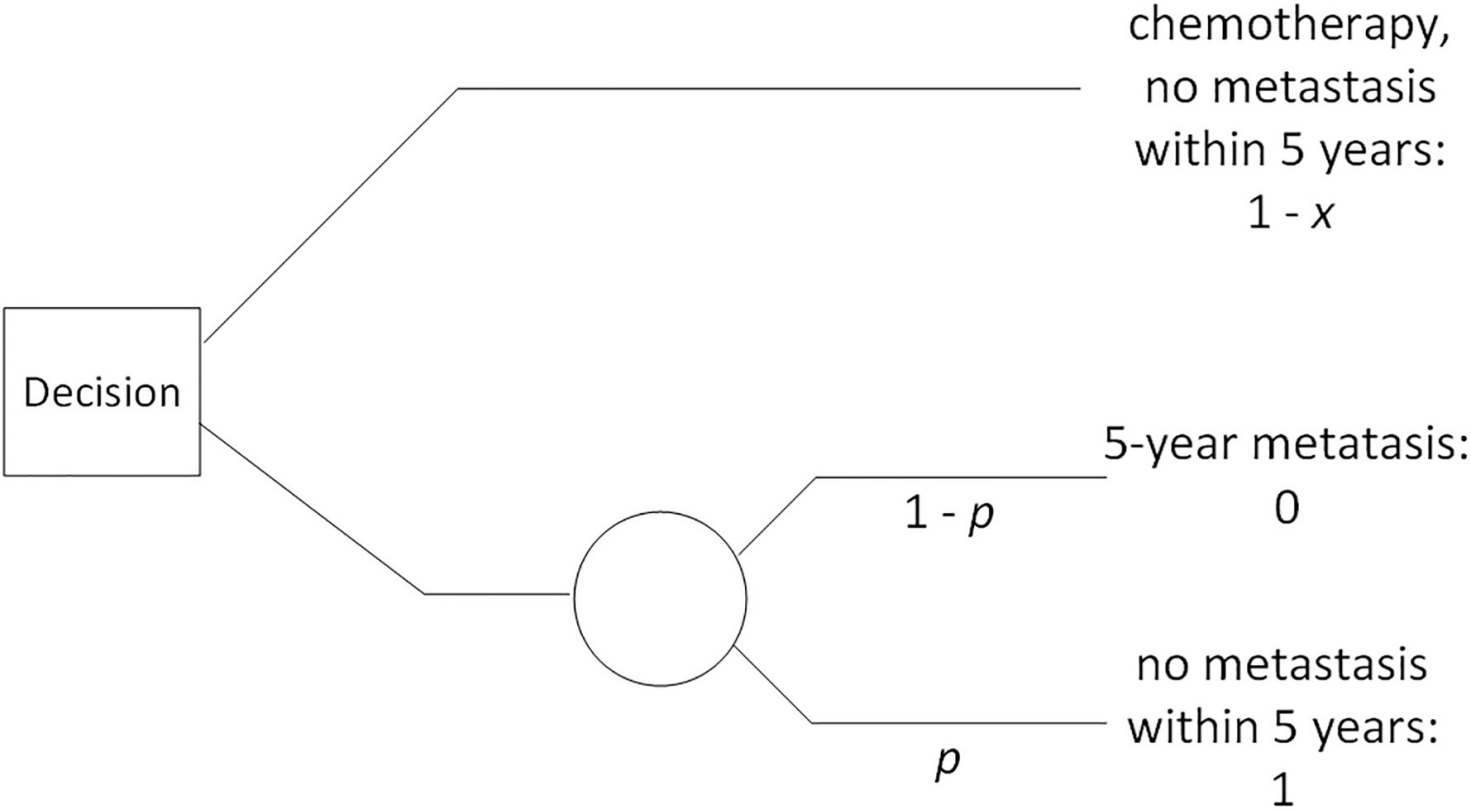
The standard gamble when a patient is deciding whether to take chemotherapy to avoid 5-year metastasis.

Next we would employ the standard gamble shown in Fig 7 with p = 0.9. We have set the value of metastasizing in 5 years to 0 and the value of not metastasizing in 5 years to 1. The value of taking chemotherapy and not metastasizing is then a value in between 0 and 1, and we have set it to 1 –*x*. Mary is indifferent between the top decision to have chemotherapy and the bottom decision to not have chemotherapy and *gamble* with probability *p* (where *p* = 0.9) of not metastasizing. To determine the value of *x*, we therefore set the certain value of the outcome on the top equal to the expected value of the outcome on the bottom, and solve for *x* as follows:

$$\begin{array}{ll} 1-x & =0\times \left(1–p\right)+1\times p\\ & =p=0.9. \end{array}$$

**Fig 8:**
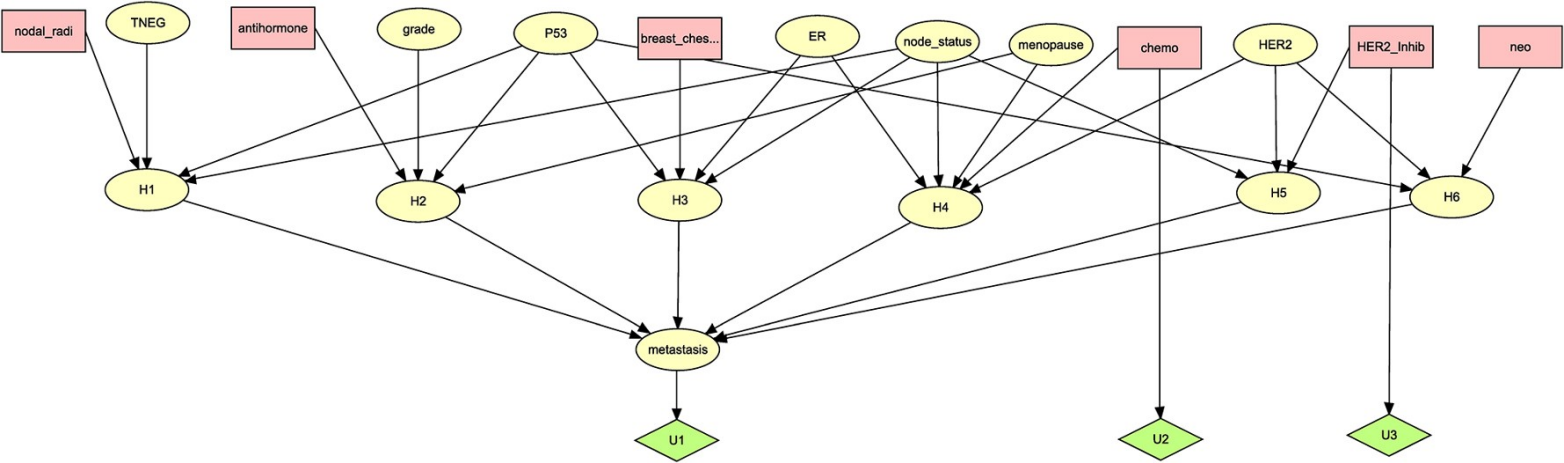
The DPAC system in Fig 6 with utility nodes for *chemo* and *HER2_inhib* added.

Solving for *x*, we obtain *x* = 1–0.9 = 0.1. This means that having chemotherapy diminishes the utility of her outcome by 0.1. To incorporate this into the DPAC system in Fig 6, we need only add a utility node with an edge from the *chemo* decision, and give that utility node value 0 if *chemo* is *no* and value -0.1 if *chemo* is *yes*. We can add such a utility node for any treatment that could have a negative effect on quality of life. Fig 8 shows the DPAC system in Fig 6 with utility nodes for *chemo* and *HER2_inhib* added.

## Discussion

We developed DPAC, which can recommend related treatments for patients based on a composite that includes not only mutation profile but also their histopathology and clinical parameters. We learned from the LSDS-5YDM a DPAC system that recommends six different treatments for breast cancer patients based on their clinical profiles. In a 5-fold cross-validation analysis and in an analysis of the independent METABRIC dataset, patients who took the treatment advice of DPAC had, as a group, notably better outcomes than those that did not. So, we can conclude that DPAC is effective at amassing and analyzing data towards treatment recommendations.

Several of the results in our DPAC system are controversial. For example, DPAC says that chemotherapy can actually increase the probability of early metastasis for many node negative patients. The same is true for neoadjuvant therapy if the patient is not P53 negative and HER2 positive. Recent research has indicated that chemotherapy at suboptimal dose can induce metastatic properties [25], and neoadjuvant therapy can increases the risk of metastatic dissemination [26]. This controversy shows the importance of developing a conclusive version of DPAC, towards resolving the controversy, and making certain we provide patients with the best patient-specific treatment recommendations.

A complete DPAC system would include clinical data and genomic data such as the 21-Gene Expression Assay [23], and take into account negative side effects of treatments using QALE. Furthermore, to have confidence in the model, it needs to be learned from at least several datasets like the one we developed. This can accomplished by using a meta-analysis approach [27] when learning a DPAC system. These EHR data exist at medical facilities throughout the world. However, the databases are usually messy and unstructured. This is a common problem with ‘big data’. While creating the LSDS-5YDM, we developed methods for handling such data, as described [28]. In future research we plan to develop another dataset from the University of Pittsburgh EHR datasets, and learn a DPAC system from the LSDS-5YDM and that dataset together, which will test the meta-analysis approach.

Our master LSDS actually contains data on four different types of chemotherapy (alkylating agents, anthracyclines, antimetabolites, antitubulin) and three types of antihormone agents (anastrozole, tamoxifen, letrozole). Although 6726 records seems like a large number, it is barely sufficient to meet the needs of a BN-based system like we developed. So we grouped the therapies into chemotherapy and antihormone. With more extensive data, individual therapies could be included in the model with the potential for more precise decision making.

When learning from retrospective data, we must be careful of hidden common causes, which can confound the results. In the case of a DPAC system, there could be a common cause of a treatment and metastasis, which is not included in the interaction for that treatment. For, example, large tumor size could both cause the doctor to prescribe chemotherapy and lead to early metastasis, which could make chemotherapy appear to cause metastasis. As we noted earlier, we used the GES algorithm to check and make sure that no variable in the dataset was such a common cause. However, there could be some hidden variables or group of variables, which are not in the dataset, which could affect the physician’s treatment decision and affect metastasis. This could simply be the physician’s subjective summary of the patient, based on years of practice. Such variables could account for why nodal therapy apparently was detrimental for most patients. Before a DPAC system could be used in practice, it should finally be evaluated in a case-control study.

As noted in the Results Section, a limitation of our study is that 5 year follow-up is considered insufficient in most cases of breast cancer. We chose to only investigate 5 year metastasis so that we could be confident that our time horizon would enable us to obtain accurate results that could verify the effectiveness of our algorithm. Too many patients left the study after 5 years. Furthermore, avoiding metastasis is the direct goal of treatments; so we investigated that goal rather than overall survival. Now that we have established that the algorithm is effective using a 5-fold cross-validation analysis, in future research we plan to develop a more comprehensive system that looks 10 and 15 years into the future, and that predicts overall survival, which is more standardly done. We hope to compare this system to PREDICT [29], which uses similar co-variates to ours and which performs overall survival prediction.
